# Trends and Disparities in Suicidal Thoughts and Behaviors Among an Ethno-Racially Diverse Group of Adolescents: 2013–2022

**DOI:** 10.1007/s40615-025-02431-8

**Published:** 2025-05-15

**Authors:** Eunice M. Areba, Michelle A. Mathiason, Patricia I Jewett, Lindsay A Taliaferro, Iris W Borowsky

**Affiliations:** 1 School of Nursing, University of Minnesota, Minneapolis, MN USA; 2 Division of Environmental Health Sciences, School of Public Health, University of Minnesota, Minneapolis, MN USA; 3 Department of Population Health Sciences, College of Medicine, University of Central Florida, Orlando, FL USA; 4 Division of General Pediatrics & Adolescent Health, Department of Pediatrics, Medical School, University of Minnesota, Minneapolis, MN USA

**Keywords:** Suicidal ideation, Suicide attempt, Age and ethnic and racial time trends, Adolescent, Health disparities, Race and ethnicity

## Abstract

**Objective:**

Suicide is a leading cause of death among adolescents and research on aggregated data conceals unique vulnerabilities across ethno-racial subpopulations. Adverse childhood experiences (ACEs) increase risk for suicide ideation (SI) and suicide attempts (SA), but these associations may vary across different ethno-racial groups and years.

**Method:**

Data from the 2013–2022 Minnesota Student Survey were used to assess trends in past-year SI and SA (grades 8, 9, and 11, *n* = 421,709). We described frequencies of demographics and evaluated trends using the Mantel–Haenszel tests of linear associations stratified by 12 ethno-racial groups and sex. Using logistic regression models, we assessed how SI and SA outcomes varied across ethno-racial groups when adjusting for grade and ACEs.

**Results:**

Overall, SI and SA increased among 8 th and 9 th grade female students. Female students more frequently reported SI (14.0–20.1%, *p* < 0.001) and SA (4.9–5.6%, *p* < 0.001), compared to male students (SI 6.3–8.8%; SA 1.9–2.5%, *p* < 0.001), with Black Latine, American Indian/Alaskan Native (AIAN), and multiracial students consistently reporting high rates. Rates of SA significantly increased among AIAN male and AIAN and Black female students. ACEs were strongly related to SI and SA, but time trends were not limited to changes in ACEs for most groups.

**Conclusion:**

These marked differences in SI and SA call for targeted and multipronged prevention approaches that account for shared and distinct factors across sex, developmental stages, and ethno-racial subgroups. To develop acceptable and efficacious interventions identifying amenable targets within subgroups is critical.

**Supplementary Information:**

The online version contains supplementary material available at 10.1007/s40615-025-02431-8.

## Introduction

Suicide is the second leading cause of premature death among adolescents and young adults aged 10–24 [1]. Suicide rates in this age group were stable between 2001 and 2007 and then increased 62.0% between 2007 and 2021 [1]. Between 2018 and 2021, significant changes in suicide rates from 2018 were observed as follows: rates increased among Black youth aged 10–24 (36.6%), while rates increased among those aged 25–44 who identified as Hispanic/Latine (19.4%), multiracial (20.6%), Black (22.9%), and American Indian or Alaska Native (AIAN) (33.7%). Non-Hispanic white (nHwhite) individuals were the only group with an overall 3.9% age adjusted decline in suicide deaths in the same period [2].

Suicide, or suicidal behaviors, is part of a wide classification of behaviors termed self-directed violence, and suicidal ideation (SI) is a broad term describing preoccupations or thoughts of engaging in suicide-related behavior [3]. Etiological risk factors for suicide are complex and interrelated across individual, relationship, and societal, community, or structural levels. Young people who experience suicidal thoughts, mental health concerns (e.g., depression, anxiety, conduct disorder, bipolar disorder, personality disorders), and/or substance use disorders demonstrate increased risk for suicide [4–8], with prior suicide attempts representing the strongest predictor of dying by suicide [9]. Extant research identify traumatic life experiences and interpersonal stressors, including adverse childhood experiences (ACEs), physical and cyber bullying, and problems in school and relationships (e.g., boyfriend/girlfriend, family problems) as some of the most common precipitants of deaths by suicide among adolescents [7, 10–12]. Social and political determinants in environments within which young people grow can also heighten suicide risk. For example, acculturation stressors [13], stigma related to mental health and help-seeking, harmful media depictions of self-harm [14, 15], access to lethal means (e.g., firearms) [16], and challenges emanating from traumatic events such as the COVID-19 pandemic (that compound longstanding inequities for marginalized groups) are associated with increased risk of suicidal thoughts and behaviors (STBs) among youth [17]. Structural racism, which maintains racial inequities through unequal distribution of resources (e.g., healthcare services, health insurance, school funding), opportunities, and risks, also creates environments that reinforce and nurture systemic and interpersonal experiences of prejudice and discrimination [18]. Experiences of racism also compound intrapersonal risk factors such as hopelessness and depression that in turn increase the risk for suicide.

Understanding the pathways to suicidal ideation and attempt(s) is critical in developing suicide prevention strategies. Prior studies have examined associations between childhood adversity such as child maltreatment [19] and suicidal behaviors in clinical samples of youth, adolescents in military families [20], and a statewide school-based sample [21]. Defined as highly correlated and potentially traumatic experiences during childhood (0–17 years), ACEs [22] (e.g., child maltreatment, neglect, abuse, and household substance use) can disrupt neurodevelopment [23], negatively affect self-esteem and self-regulation [24], and increase the risk of dying by suicide [25]. Youth are likely to experience more than one ACE [22, 26], and researchers have documented a graded dose–response effect of ACEs on health outcomes, including substance use, depression, and STBs, throughout the life course [27, 28], and in adolescence [29]. ACE experiences are dependent on many factors including the environments within which young people grow. Thus, they are varied across populations, and the effects of ACEs, including suicidal thoughts and behaviours (STBs), also likely vary across ethno-racial groups.

National reports indicate disparities in suicide ideation and attempt(s) and suicide rates by grade, sex, and ethno-racial groups [1, 17, 30, 31]. Data from the Centers for Disease Control and Prevention (CDC)’s 2019 [30] and the 2021 [17]. Youth Risk Behavior Surveillance survey showed that one-third of female students reported seriously considering suicide during the past 12 months, with greater prevalence observed among Black, Latine, and white female students. Black and Latine male students were more likely than white male students to report a suicide attempt that required medical treatment. However, researchers rarely disaggregate data by ethno-racial groups and subgroups, limiting our knowledge of trends within specific populations. Aggregated data conceals variability in risk and protective factors within groups and between subgroups and curtails the reach and effectiveness of surveillance and prevention strategies that are not tailored to address precipitating circumstances and risk factors for specific groups.

Across the USA, ethno-racially minoritized communities (i.e., groups currently and historically marginalized) [32], including youth, are unevenly distributed due to regional, neighborhood and school segregation patterns. Although the share of the population that identify with a race other than white has increased over time, compared to the rest of the USA (white (75.3%), Black (13.7%), Latine (19.5%), Asian (6.4%), AIAN (1.3%)] [33], the state of Minnesota is less diverse. About 23% or 1.3 million people are Minnesotans of color. This includes diverse Black (7.9%), Latine (7%), Asian (5.5%), white (77%, down 3% since 2018)), and 11 federally recognized tribes of Native American residents (2%) [34]. To identify where health inequities lie, we need to categorize ethno-racial groups accurately. One way to achieve this involves meaningful and thoughtful data disaggregation. The US Census Bureau found that over time, some historically used racial and ethnic categories do not coincide with how people identify, which can be complex. For example, Latine, Middle Eastern/North African (MENA), and multiracial groups often describe their heritage differently. These groups (or subgroups within these pan ethnic groups) are seldom the explicit focus of research studies. Researchers often include individuals who identify as MENA within a “white” group, which may differ from how some MENA individuals prefer to self-identify [35]. In 2020, about 3.5 million people in the US identified as MENA, including close to 26% of youth under 18 years [36]. MENA persons are more likely to experience economic hardship, have lower rates of home ownership [37], and have higher age-adjusted mortality risk [38] compared to white-only populations. Some MENA populations encounter additional stressors that increase their risk for poor health outcomes. For example, those who experienced high social needs (e.g., transportation barriers to healthcare, food insecurity) and fears of deportation and discrimination reported poor mental health and sleep difficulties [39–41]. Similarly, Black Latine (or Afro-Latine) populations have worse health outcomes and poorer mental health [42], including among young children [43], than white Latine individuals [44]. Therefore, using a statewide representative sample of 8 th, 9 th, and 11 th grade students, we sought to (a) present single year data of suicidal ideation (SI) and suicide attempt(s) (SA) among the MENA group, as these data were first collected in 2022; (b) assess trends in SI and SA from 2013 to 2022, stratified by sex and ethno-racial groups, with a particular emphasis on Black subpopulations; and (c) examine how SI and SA outcomes time trends change among ethno-racial groups when adjusted for ACEs. We hypothesized that (a) SI and SA rates would increase across the years, especially among some ethno-racially minoritized groups, such as Black and female students; (b) that all youth would experience an increase in SI but some male and ethno-racially minoritized student groups would report more attempts compared to female and white students, respectively, across the years.

## Methods

### Study Design and Data Collection

The Minnesota Student Survey (MSS) is a voluntary triennial cross-sectional statewide survey administered by school districts and state agencies in public schools since 1989. This anonymous survey provides information on youth at the state, county, and school district levels in Minnesota and examines risk and protective factors and behaviors. The MSS includes questions on multiple topics, such as risk behaviors (e.g., vaping, tobacco, alcohol, and other drug use), healthcare, nutrition, sexual activity, and protective factors, positive behaviors and connections to family, school, and community. Questions about some topics, including STBs and non-suicidal self-injury, are not part of the 5 th grade version of the survey; thus, our sample comprises only 8 th, 9 th, and 11 th graders. Parents were given the opportunity to decline their child’s participation in the survey prior to administration. Except for online students, surveys were completed during school time. As part of the data cleaning process and to ensure responses were valid, the Department of Health removed surveys with highly inconsistent responses or those with obvious exaggeration patterns prior to sharing the data with our research team. On average, across all grades, the survey took approximately 25 minutes to complete each year (Bob Kuziej, Community Health Division, Minnesota Department of Health, e-mail communication, June 5 th, 2023). Across 330 public school districts, school participation ranged from 70% in 2022 to 85% in 2013. Our analytic sample consisted of 8 th, 9 th, and 11 th graders who self-reported their ethno-racial identity, SI and SA (*n* = 421, 709) in the 2013, 2016, 2019, and 2022 MSS surveys. The MSS data is not weighted and is only administered in a single state; thus, the population-based data do not represent the US population. The Institutional Review Board at the University of Minnesota exempted the study from full board review because these data were anonymous.

#### Measures

Two items on race and ethnicity/cultural heritage were used to create 12 ethno-racial groups: American Indian/Alaskan Native (AIAN, alone or with other races, except Black), Black American Indian/Alaskan Native; Black Latine; Somali; Black/African American (Black only, Black/white, Black/other); Hmong; Asian (Asian only, Asian/white); Latine (only or white/Latine); Native Hawaiian/Pacific Islander (NHPI alone, NHPI/Asian, NHPI/white); non-Hispanic white (nHwhite); multiracial (3 or more races), MENA (non-Black, only in the 2022 survey wave). Ethno-racial categorization was informed by (a) differences in suicide thoughts and behaviors (STBs) as observed across subpopulations, (b) Minnesota demographics, including large more recent immigrant and refugee populations (e.g., Somali and Hmong), and (c) sample size limitations. Specifically, although we purposefully disaggregated ethno-racial groups into smaller subgroups, this process can limit the types of analysis that can be performed on groups that have a smaller share of the population, for example, Black subgroups, and the MENA group for which we had data for only 1 year. Additionally, because suicide attempts are rare compared to suicidal thoughts, we limited our samples to at least *n* = 15 students in a group per outcome in a survey year, to avoid unintentionally unmasking students. We prioritized marginalized identities when students identified with multiple groups (e.g., prioritizing AIAN over other races, except Black). This is because both populations have experienced significant historical injustices, which have resulted in continued intergenerational negative effects, including suicide. Additionally, Black youth demonstrate the greatest increase in STBs, while AIAN youth have consistently experienced the highest rates of STBs of any group.

Two items assessed past-year suicidal thoughts and behavior: “Have you ever seriously considered attempting suicide?” and “Have you ever actually attempted suicide? Responses were dichotomized to “no”/“yes, more than a year ago” versus “yes, during the last year.”

Demographic variables included sex (male, female) and grade (8 th, 9 th, and 11 th grades).

Based on our prior work [45], where we found that adverse childhood experiences (ACEs) were strongly associated with suicide attempts and suicidal ideation, we additionally ran a regression analysis to estimate relative odds of SI and SA comparing survey years. We adjusted for ACEs in our analyses to see if our observed unadjusted time trends were attenuated when accounting for ACEs. ACEs were assessed using the following items, (1) *verbal abuse* (Does a parent or other adult in your home regularly swear at you, insult you or put you down?); (2) *physical abuse* (Has a parent or other adult in your household ever hit, beat, kicked or physically hurt you in any way?); (3) *familial sexual abuse* (Has any relative/family member ever pressured, tricked, or forced you to do something sexual or done something sexual to you?); and *non-familial sexual abuse* (Has anyone who was not a relative/family member ever pressured, tricked, or forced you to do something sexual or done something sexual to you against your wishes?) (prior to 2019, sexual abuse was operationalized differently, …”if anyone older or stronger ever touched you or had you touch them sexually”); (4) witnessing parental/other adult *intimate partner violence*; and (5) *household substance use* (Do you live with anyone who drinks too much alcohol?) and (6) Do you live with anyone who uses illegal drugs or abuses prescription drugs?); (7) past year *housing instability* (“… have you stayed in a shelter, somewhere not intended as a place to live, or someone else’s home because you had no other place to stay?”); (7) *parental incarceration* (“Have any of your parents or guardians ever been in jail or prison?”); (8) past 30 days *food insecurity* (“… have you had to skip meals because your family did not have enough money to buy food?”). Responses to ACE items were yes versus no, and we categorized ACEs into 0, 1–3, ≥ 4, and missing.

#### Statistical Analysis

Trends, stratified by ethno-racial group and sex (male/female), were evaluated using Mantel–Haenszel tests of linear associations for past-year SI and SA, when all four survey years were available and had *at least 15 cases* per survey year. In instances where cases were fewer than 15, data were combined across MSS waves (e.g., Black subgroups, NHPI, and multiracial groups; refer to Supplemental Table 2). We tabulated frequencies of exposure to adversity by ethno-racial group and sex and graphed trends across ethno-racial groups in bar charts. We compared Black subgroups and MENA using multiple comparisons Tukey–Kramer adjustment between groups with 2019 and 2022 data combined (Table 2). To further assess trends in SI and SA, we stratified multiple logistic regression models by ethno-racial group and sex, adjusted by grade and ACE. Using 2013 as a reference, we compared odds of SI and SA in all survey years across ethno-racial groups. We report odds ratios (OR) and 95% confidence intervals (CI). To account for small sample sizes in some groups, each regression model was *Firth*-adjusted [46].

## Results

**Fig. 1:**
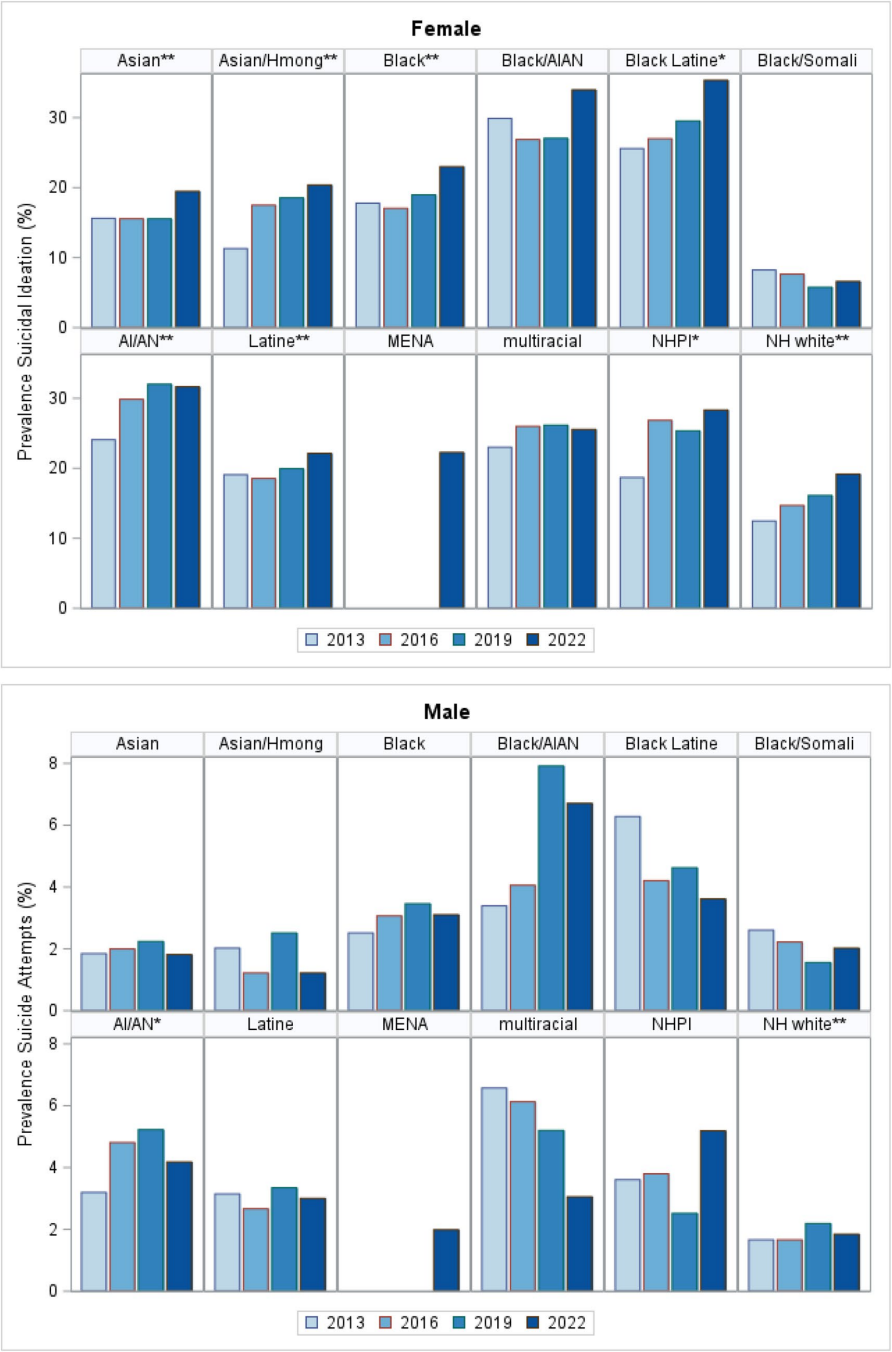
Prevalence of suicidal ideation by sex and ethno-racial groups, 2013–2022 Minnesota Student Surveys (MSS). NHPI (Native Hawaiian/Pacific Islander), AI/AN (American Indian/Alaskan Native), nHwhite (non-Hispanic white), MENA (Middle Eastern/North African). Linear trends in the prevalence of suicidal thoughts. *p < 0.05; **p < 0.001

**Fig. 2:**
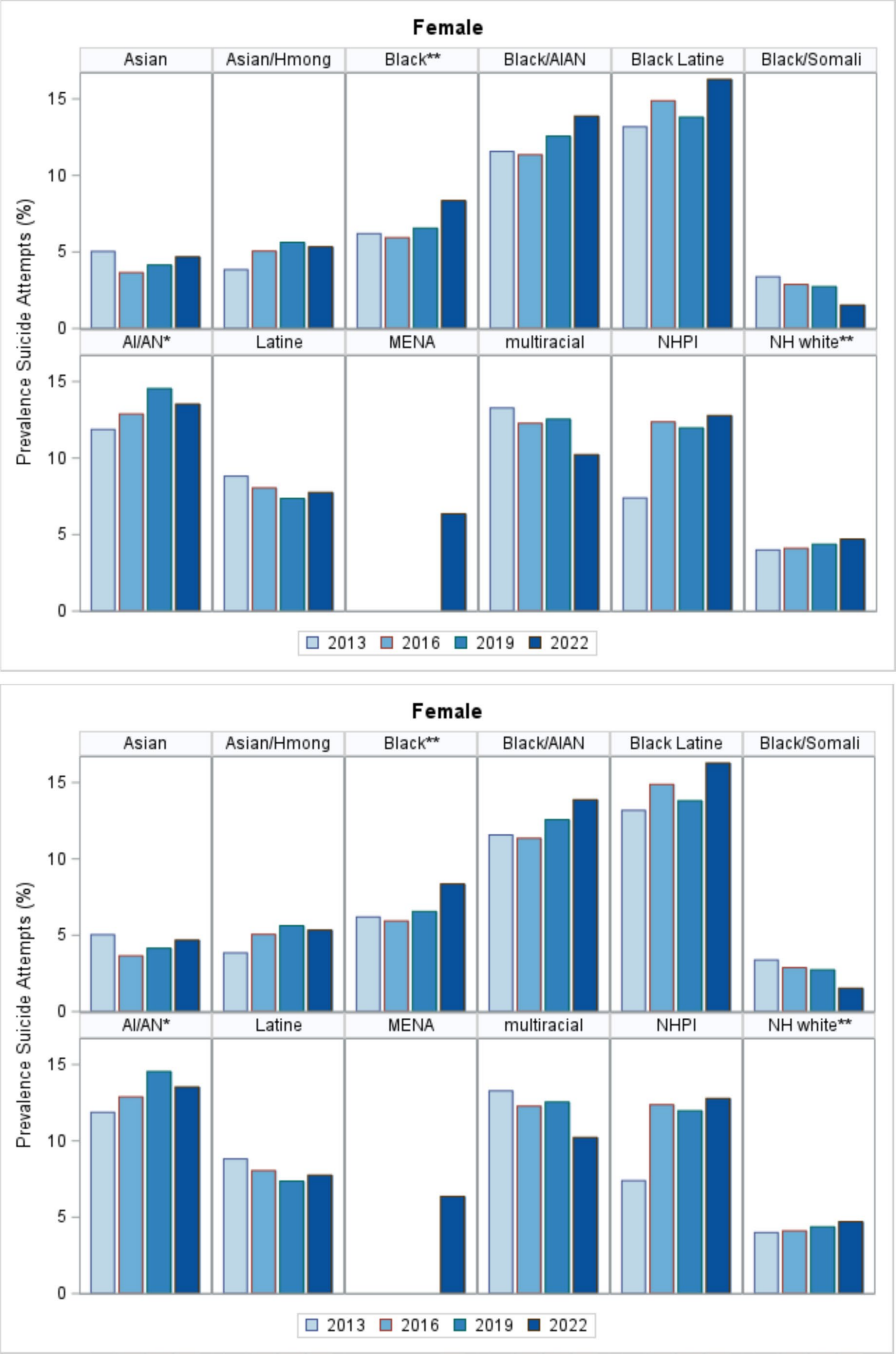
Prevalence of suicide attempts by sex and ethno-racial groups, 2013–2022 Minnesota Student Surveys (MSS). NHPI (Native Hawaiian/Pacific Islander), AI/AN (American Indian/Alaskan Native), nHwhite (non-Hispanic white), MENA (Middle Eastern/North African). *p < 0.05; **p < 0.001

Male (49%) and female (51%) students were evenly distributed across ethno-racial groups, and there were more students in 8^th^ grade compared to 11^th^ grade across groups, except for Hmong students whose numbers were stable in 8^th^ through 11^th^ grade (Table 1). Female students in both 8 th (23.4%, 95% CI 22.3%, 24.5%) and 9 th grade (22.4%, 95% CI 21.2%, 23.5%) were more likely to report SI, compared to female students in 11 th grade (19.9%, CI, 18.5%, 21.3%), but there were no differences in SI among male students across all grades. Similarly, female students reported the highest rates of SA in 8 th grade (9.2%, 95% CI 8.5%, 9.9%) compared to 11 th grade (5.5%, 95% CI 4.6%, 6.4%). Male SA rates were not significantly different across grades (Supplemental Fig. 1). From 2013 to 2022, female students more frequently reported SI (14.0–20.1%) and SA (4.9–5.6%), compared to male students (SI 6.3–8.8%; SA 1.9–2.5%) (Figs. 1 and 2, Supplemental Tables 1 and 2). Rates varied and increased over time across ethno-racial groups. Black Latine, AIAN, and NHPI students consistently reported the highest rates of SI and SA. From 2013 to 2022, SI rates significantly increased among male students (e.g., Black (6.7–9.2%), Asian (6.7–9.5%), nHwhite (5.9–8.0%)) and female students (e.g., Black Latine (25.6–35.3%), Black (17.8–23.0%), Hmong (11.3–20.4%), and AIAN (24.1–31.6%)) (Fig. 1, Supplemental Table 1).

Overall, female students experienced a significant increase in SA rates from 2013 until 2022 (4.9–5.6%) and specifically among Black (6.2–8.4%) and AIAN female students (11.9–13.5%) (Fig. 2, Supplemental Table 2). Although not significant, SA rates also increased among Black male students, and Black Latine, Black/AIAN, and NHPI female students. SA rates also increased significantly among AIAN male students (3.2–4.2%) over the same period.

Somali adolescents consistently reported the lowest rates of both SA and SI (Figs. 1 and 2, Supplemental Tables 1 and 2). Rates of SI and SA among Somali male students were significantly lower than in other Black male and female subgroups. Among male students, Black/AIAN students had the highest SA rates, and their rates were significantly higher than Black Somali, Black Latine, and MENA males. Black/AIAN and Black Latine female students had significantly higher rates of SI and SA compared to other Black subgroups and MENA (Table 2).

In the logistic regression models by survey year (unadjusted: accounting for grade only; adjusted: accounting for grade and number of ACEs, Supplemental Tables 3 and 4), associations of each survey year with SI and SA were similar across ethno-racial groups and sex. Some examples of almost identical associations, with 2013 as the reference year included, SI, Black, male, 2016 unadjusted OR 1.22, 95% CI 1.01–1.48 versus adjusted OR 1.30, 95% CI 1.07–1.58; SI, AIAN, male, 2022 unadjusted OR 1.45, 95% CI 1.17–1.79 versus adjusted OR 1.44, 95% CI 1.16–1.78; and SA, Black, female, 2022 unadjusted OR 1.37, 95% CI 1.12–1.67 versus adjusted OR 1.48, 95% CI 1.21–1.82. There were some exceptions when associations of survey year with the outcomes were attenuated, e.g., SA, NH white, female, 2022 unadjusted OR 1.18, 95% CI 1.10–1.27 versus adjusted OR 1.01, 95% CI 0.94–1.09.

**Table 1:** Sample characteristics across ethno-racial groups, 2013–2022 Minnesota Student Surveys (MSS) (*n* = 421,709)

	Latine	Black Somali	Black Latine	Black AIAN	Black	Hmong	Asian	NHPI	AI/AN	MENA	nHwhite*	multiracial^
***N* (%)**	31,159	6134	1827	2972	25,260	9534	20,992	2115	17,083	585	301,918	2130
**Grade**												
8 (*n* = 152,443)	38.6	41.7	43.6	40.8	40.2	33.5	37.8	39.2	41.2	42.2	35.0	42.3
9 (*n* = 148,307)	35.3	34.3	35.3	34.7	34.5	35.3	35.2	34.8	37.5	33.7	35.1	35.1
11 (*n* = 120,959)	26.1	24.0	21.2	24.5	25.3	31.2	27.1	26.1	21.4	24.1	29.9	22.6
**Sex at birth**												
Male (*n* = 208,551)	48.7	47.00	46.8	41.6	49.2	51.1	48.9	52.5	51.8	51.6	49.5	48.6
Female (*n* = 213,158)	51.3	53.00	53.2	58.4	50.8	48.9	51.1	47.5	48.2	48.4	50.5	51.4
**ACE, male**												
0 (*n* = 133,885)	55.8	72.1	38.4	32.4	47.2	56.9	64.5	51.6	43.6	70.2	68.4	44.6
1–3 (*n* = 59,056)	34.5	15.4	48.0	48.8	39.8	33.8	28.8	36.4	44.9	24.2	25.4	39.9
4 or more (*n* = 5729)	3.4	2.6	7.5	9.6	4.5	2.3	2.1	5.7	6.1	2.0	2.3	7.6
Missing (*n* = 9881)	6.3	10.0	6.2	9.2	8.5	7.0	4.7	6.4	5.5	3.6	4.0	7.8
**ACE, female**												
0 (*n* = 125,703)	47.1	73.9	31.6	27.1	43.1	52.6	62.8	44.7	33.2	52.7	63.4	37.7
1–3 (*n* = 67,229)	38.5	15.9	47.3	49.4	42.1	36.0	29.8	39.9	47.0	35.0	29.0	40.9
4 or more (*n* = 11,704)	8.3	2.2	14.0	17.7	7.6	4.7	3.4	10.7	14.5	5.7	4.5	15.0
Missing (*n* = 8522)	6.1	8.0	7.1	5.8	7.3	6.7	4.0	4.7	5.3	6.7	3.2	6.4

^Students who checked 3 or more racial groups

*Non-Hispanic white

**Table 2:** Estimated past-year prevalence of suicidal ideation and suicide attempt among Black and MENA groups

	Male			Female	
	Estimate (95% CI)			Estimate (95% CI)	
Suicidal ideations	Black Somali	3.4%	(2.0%, 4.8%)^**∆,∫,◊**^	6.1%	(4.4%, 7.9%)^∆,∫,◊,*^
	Black Latine	10.9%	(8.0%, 13.9%)^**∂**^	32.5%	(28.8%, 36.1%)^∂,◊,*^
	Black AIAN	15.1%	(12.4%, 17.8%)^∂,◊,*^	29.6%	(26.4%, 32.7%)^**∂,◊**^
	Black	9.2%	(8.5%, 9.9%)^**∫,∂**^	20.8%	(19.9%, 21.8%)^**∂,∆,∫**^
	MENA	6.0%	(2.8%, 9.1%)^**∫**^	22.3%	(17.8%, 26.8%)^**∂,∆**^
Suicide attempts	Black Somali	1.8%	(0.9%, 2.7%)^**∫,◊**^	2.2%	(1.0%, 3.3%)^**∆,∫,◊**^
	Black Latine	4.1%	(2.3%, 6.0%)	15.1%	(12.6%, 17.5%)^∂,◊,*^
	Black AIAN	7.4%	(5.7%, 9.1%)^∂,◊,*^	13.0%	(11.0%, 15.1%)^∂,◊,*^
	Black	3.3%	(2.9%, 3.7%)^**∂,∫**^	7.4%	(6.8%, 8.0%)^**∂,∆,∫**^
	MENA	SP	SP	6.4%	(3.4%, 9.3%)^**∫,∆**^

Multiple pairwise comparison tests with Tukey–Kramer adjustment, statistically significant differences to group depicted by the following symbols: Black/Somali, ∂; Black/Latine, ∆; Black/AIAN, ∫; Black, ◊; Middle Eastern and North African (MENA), *

*SP*, suppressed

## Discussion

Our findings are consistent with previous population-based studies demonstrating that SI and SA are increasing among adolescents [1, 17, 30, 31], especially among some ethno-racial groups such as Black adolescents, and that AIAN youth report the highest rates of SI and SA. Our findings highlight the importance of examining subgroups within pan-ethnic groups, such as “Black/AA” or “Asian” and MENA as a distinct group that is separate from the nHwhite group. There were significant linear trends in SI among female students in 8^th^ and 9^th^ grade and SA rates increased among 8^th^ graders between 2019 and 2022. Among male students, SI rates increased between 2013 and 2019 and then stabilized in 2019 and 2022. Our results indicate that age, sex, and ethno-racial group are important factors in developing prevention strategies.

More information is needed on groups rarely centered in research, despite alarming rates of STBs, e.g., Black Latine, NHPI, multiracial, and AIAN youth. Specific data can better inform surveillance and direct tailored prevention efforts. For example, by examining risk and protective factors across subgroups with low SI and SA rates, e.g., Somali youth, and those with higher rates, such as Black AIAN and Black Latine youth, we can develop interventions that are more likely to be efficacious. By reducing the effects of unique stressors in these subgroups. Notably, these three groups with vastly diverging rates of SI and SA (i.e., Somali, Black AIAN, and Black Latine youth) are often lumped together and examined as one group, Black/African American.

Prior research found steady increases in SA among Black adolescents from 1991 to 2017, including suicide attempts resulting in injuries that required medical attention, among Black males [31]. From 2003 to 2017, a significant increase in suicide deaths was also observed among Black adolescents [7]. SI and SA rates among Black adolescents continued to increase in 2021 [17]. A recent investigation into Black youth suicide found that suicide rates were highest in adolescents aged 15–17, while Black female students in the 12–14-year-old age group experienced the highest annual increase in suicide rates [7]. Prior studies also point to Black youth using more lethal means in suicide attempts, such as firearms [47], and illustrate the gravity and impact of the stressors Black adolescents contend with. These concerning findings underscore the need for more nuanced data on suicide in Black youth subgroups.

We ran regression models with survey year as the predictor, unadjusted and adjusted for the number of ACEs. The rationale was that in our previous work, we found strong associations of increasing ACE exposures with more frequent SI and SA. Therefore, we expected that the inclusion of ACEs would attenuate some of the unadjusted time trends, with the underlying idea that ACE exposures might have increased over time. However, with some exceptions, we found no attenuation of the time trends after adjusting for number of ACEs, suggesting that the time trends were driven by factors unrelated to changes in ACEs assessed in this study. Examining which other underlying factors that changed over time were driving the observed time trends should be the subject of future studies.

The recent COVID-19 pandemic compounded long standing inequities and resulted in the loss of parents or primary caregivers of close to 200,000 children in the USA, most of whom were Black and AIAN [48]. Further, Asian youth reported anti-Asian sentiment (degrading name calling and actions because of their cultural heritage) that was linked to the origins of the causative virus, SARS-CoV- 2 [49]. Incidents of youth dying by suicide because of being bullied for their family’s immigration status have also been reported [50]. Xenophobia and similar “othering” ideologies, such as islamophobia and racism, do not occur in a vacuum. Spurred by political rhetoric and societal level sentiments that dehumanize ethno-racially minoritized communities, youth with intersecting marginalized identities, such as those related to immigration status, living in mixed status families and sexuality and gender, class and ability, experience unique stressors that significantly contribute to the ongoing youth mental health crisis [51]. Environments that deprive young people of opportunities for healthy development (e.g., economic hardship, discrimination, racism) are rife with adverse experiences that may in turn, inhibit adaptive coping, compromise emotional regulation, and facilitate spontaneous actions that are life limiting such as SI and SA [24, 52, 53].

## Future Directions

We observed marked differences in SI and SA across ethno-racial groups and highlighted great variability within Black youth subgroups. These differences in SI and SA call for targeted and multipronged prevention approaches that account for group specific risk and protective factors within schools, households, and society; how these factors interact with different stages of development and age; and the effects of institutional policies and social norms on minoritized identities (e.g., race, ethnicity, gender, sex, religion). Such approaches should include fostering meaningful social connections, affirming adolescents’ ethno-racial identity [54], increasing economic supports, and preventing and mitigating the effects of ACEs (e.g., through home visiting programs [55] during vulnerable periods in life and nurturing supportive adult relationships). Other strategies include training school personnel in identifying and responding to suicide risk, referring students to pediatric primary and mental health services, and decreasing mental health stigma by working with culturally acceptable community-based organizations (e.g., religious and cultural affinity organizations). It is important that there is congruence in the ethno-racial backgrounds and lived experiences of personnel within these organizations that serve ethno-racially minoritized youth.

In comparing nationwide CDC data and statewide MSS data, Wiglesworth et al. [56] contend that better comprehension of the heterogeneity within AIAN and Black populations is essential in understanding suicide risk in both groups. The researchers further suggested considering regional differences and subpopulation characteristics, such as nativity and migration status and examining systemic and institutional racism and trauma.

Our study has strengths and limitations. The MSS is a large state population-based dataset that enables examining SI and SA across ethno-racial groups. Like other epidemiological surveys, single-item measures of SI and SA in the MSS provide incomplete information about the nature of STBs, although they have high face validity and reduce participant burden. Results from population-based surveys such as the CDC’s YRBS and MSS facilitate surveillance of suicide risk, protective factors (assessed in MSS), and prevalence of SI and SA. This information can be used to enhance and guide prevention efforts, including screening guidelines in clinical settings where in-depth validated suicide risk screeners (e.g., the Columbia Suicide Severity Rating scale [C-SSRS]) are recommended to identify adolescents at high risk of suicide and in need of further assessment. We acknowledge that some students may have been misclassified. While our study expands on ethno-racial groupings used in most research, our 12 ethno-racial groups are not exhaustive of all ethno-racial groups. In 2022, school districts’ participation decreased, this was likely due to challenges posed by the COVID-19 pandemic and accompanying political and social discourse that prompted increased distrust of public school personnel, public health and government officials. Data on MENA students were collected for the first time in 2022, and thus, we were not able to assess trends for this group. Additionally, the demographics of Minnesota may not reflect the national ethno-racial makeup, and thus, findings are not widely generalizable. Future studies should also incorporate important demographic variables such as sexual identity and gender identity to better contextualize trends in SI and SA.

## Conclusion

From 2013 to 2022, the prevalence of SI and SA increased among some demographic groups and was stable among other groups. The increased prevalence of SI and SA among female students overall (with highest increases among Black subgroups, except Somali female students) and NHPI and Asian male students indicate important disparities that require further investigations. Suicide is multifactorial with individual-, community-, and societal-level characteristics, including ACEs contributing to differences in SI and SA between female and male students, ethno-racial groups, grade levels, and developmental stages. Identifying suicide risk and protective factors across and within ethno-racial subgroups that are usually lumped together (e.g., Black/AA, MENA) remains imperative to identify shared and distinct mechanisms underlying suicide risk and protection that can inform culturally responsive and tailored prevention interventions. Prevention efforts must account for differences observed by sex, age and ethno-racial group.

## Supplementary Information

Below is the link to the electronic supplementary material. 

ESM1 (DOCX 18.6 KB) 

ESM2 (DOCX 17.2 KB) 

ESM3 (DOCX 34.6 KB) 

ESM4 (DOCX 72.9 KB)

## Data Availability

N/A.

## Code Availability

N/A.
