# Supplementary material for: Trends and Disparities in Suicidal Thoughts and Behaviors Among an Ethno-Racially Diverse Group of Adolescents: 2013–2022
Source: J Racial Ethn Health Disparities. 2025 May 15;13(3):2452–63. doi: 10.1007/s40615-025-02431-8 (PMC13157415; doi:10.1007/s40615-025-02431-8)
Supplement: Supplementary file 1 — (DOCX 18.6 KB) [file 40615_2025_2431_MOESM1_ESM.docx]

| **Supplemental Table 1.** Prevalence of Past Year Suicidal Ideation Across Ethno-Racial Groups by Sex, n (%), 2013-2022 Minnesota Student Survey (MSS) | | | | | | | | | |
| --- | --- | --- | --- | --- | --- | --- | --- | --- | --- |
| **Male (n=207,874)** | | | | | | | | | |
|  | **2013** | | **2016** | | **2019** | | **2022** | | P-values |
| Latino | **222** | **7.3%** | **321** | **7.4%** | **410** | **9.7%** | **311** | **8.7%** | **<.001** |
| Black/Somali | 25 | 5.0% | 37 | 4.3% | 27 | 3.3% | 25 | 3.6% | 0.138 |
| Black/Latino | 24 | 11.6% | 29 | 9.4% | 17 | 9.8% | 20 | 12.0% | 0.862 |
| Black/AIAN | 49 | 11.9% | 53 | 12.7% | 37 | 15.4% | 24 | 14.6% | 0.201 |
| Black | **196** | **6.7%** | **279** | **8.1%** | **307** | **9.2%** | **250** | **9.2%** | **<.001** |
| Asian/Hmong | 92 | 6.7% | 112 | 7.2% | 106 | 8.9% | 52 | 7.0% | 0.236 |
| Asian | **135** | **6.7%** | **197** | **7.4%** | **277** | **9.4%** | **251** | **9.5%** | **<.001** |
| NHPI | 25 | 8.2% | 34 | 10.8% | 31 | 11.2% | 29 | 13.7% | 0.053 |
| AI/AN | **222** | **9.0%** | **345** | **11.0%** | **238** | **13.1%** | **176** | **12.5%** | **<.001** |
| NH white | **2405** | **5.9%** | **2827** | **6.8%** | **3231** | **8.4%** | **2290** | **8.0%** | **<.001** |
| MENA | N/A | N/A | N/A | N/A | N/A | N/A | 18 | 6.0% | NA |
| Multiracial | 26 | 12.2% | 42 | 13.6% | 34 | 13.6% | 33 | 12.6% | 0.944 |
| Total | 3421 | 6.3% | 4276 | 7.3% | 4715 | 8.8% | 3479 | 8.4% | **<.001** |
| **Female (n=212,832)** | | | | | | | | | |
| Latina | 593 | 19.1% | 827 | 18.5% | 913 | 19.9% | 848 | 22.1% | **<.001** |
| Black/Somali | 39 | 8.2% | 69 | 7.6% | 59 | 5.8% | 56 | 6.6% | 0.127 |
| Black/Latina | **66** | **25.6%** | **78** | **27.0%** | **62** | **29.5%** | **76** | **35.3%** | **0.017** |
| Black/AIAN | 168 | 29.9% | 161 | 26.9% | 99 | 27.0% | 71 | 34.0% | 0.626 |
| Black | **511** | **17.8%** | **606** | **17.0%** | **657** | **19.0%** | **677** | **23.0%** | **<.001** |
| Asian/Hmong | **141** | **11.3%** | **270** | **17.5%** | **211** | **18.5%** | **149** | **20.4%** | **<.001** |
| Asian | **338** | **15.6%** | **426** | **15.6%** | **491** | **15.5%** | **520** | **19.5%** | **<.001** |
| NHPI | **48** | **18.7%** | **26.9** | **27.0%** | **72** | **25.4%** | **51** | **28.3%** | **0.032** |
| AI/AN | **548** | **24.1%** | **853** | **29.9%** | **590** | **32.0%** | **400** | **31.6%** | **<.001** |
| NH white | **5131** | **12.5%** | **6127** | **14.7%** | **6446** | **16.1%** | **5655** | **19.2%** | **<.001** |
| MENA | N/A | N/A | N/A | N/A | N/A | N/A | 63 | 22.3% | NA |
| Multiracial | 52 | 23.0% | 72 | 26.0% | 73 | 26.2% | 80 | 25.6% | 0.561 |
| Total | 7635 | 14.0% | 9565 | 16.2% | 9673 | 17.2% | 8646 | 20.1% | **<.001** |
| **^** Students who checked 3 or more racial groups. P-values are for linear trends across the 4 surveys  NHPI [Native Hawaiian/Pacific Islander], AI/AN [American Indian/Alaskan Native], nHwhite [non-Hispanic white], MENA [Middle Eastern/ North African]  *Data were collected on MENA for the first time in 2022. | | | | | | | | | |
