# Supplementary material for: Trends and Disparities in Suicidal Thoughts and Behaviors Among an Ethno-Racially Diverse Group of Adolescents: 2013–2022
Source: J Racial Ethn Health Disparities. 2025 May 15;13(3):2452–63. doi: 10.1007/s40615-025-02431-8 (PMC13157415; doi:10.1007/s40615-025-02431-8)
Supplement: Supplementary file 2 — (DOCX 17.2 KB) [file 40615_2025_2431_MOESM2_ESM.docx]

| **Supplemental Table 2.** Prevalence of Past Year Suicide Attempts Across Ethno-racial Groups By Sex, n (%), 2013-2022 Minnesota Student Survey | | | | | | | | | |
| --- | --- | --- | --- | --- | --- | --- | --- | --- | --- |
| **Male (n=207,943)** | | | | | | | | | |
|  | **2013** | | **2016** | | **2019** | | **2022** | | P-values |
| Latino | 96 | 3.1% | 116 | 2.7% | 141 | 3.3% | 107 | 3.0% | 0.726 |
| Black/Somali^∆^ | 32 |  | 2.4% | | 27 | | 1.8% | | NA |
| Black/Latino^∆^ | 26 | | 5.0% | | 14 | | 4.1% | | NA |
| Black/AIAN^∆^ | 31 | | 3.7% | | 30 | | 7.4% | | NA |
| Black | 73 | 2.5% | 106 | 3.1% | 116 | 3.5% | 84 | 3.1% | 0.119 |
| Asian/Hmong | 28 | 2.0% | 19 | 1.2% | 39 | | 2.0% | | NA |
| Asian | 37 | 1.8% | 53 | 2.0% | 66 | 2.2% | 48 | 1.8% | 0.959 |
| NHPI | 23 | | 3.7% | | 18 | | 3.7% | | NA |
| AI/AN | **79** | **3.2%** | **151** | **4.8%** | **95** | **5.2%** | **59** | **4.2%** | **0.044** |
| NH white | **681** | **1.7%** | **688** | **1.7%** | **844** | **2.2%** | **525** | **1.8%** | **<.001** |
| MENA |  |  |  |  |  |  | SP* | 2.0% | **NA** |
| Multiracial | 33 | | 6.3% | | 18 | | 4.1% | | NA |
| Total | **1059** | **1.9%** | **1213** | **2.1%** | **1352** | **2.5%** | **888** | **2.2%** | **<.001** |
| **Female (n=214,142)** | | | | | | | | | |
| Latina | 274 | 8.8% | 359 | 8.1% | 337 | 7.4% | 297 | 7.8% | 0.062 |
| Black/Somali^∆^ | 42 |  | 3.1% |  | 41 |  | 2.2% |  | NA |
| Black/Latina | 34 | 13.2% | 43 | 14.9% | 29 | 13.8% | 35 | 16.3% | 0.428 |
| Black/AIAN | 65 | 11.6% | 68 | 11.4% | 46 | 12.6% | 29 | 13.9% | 0.360 |
| Black | **178** | **6.2%** | **211** | **5.9%** | **227** | **6.6%** | **246** | **8.4%** | **<.001** |
| Asian/Hmong | 48 | 3.8% | 78 | 5.1% | 64 | 5.6% | 39 | 5.3% | 0.069 |
| Asian | 109 | 5.0% | 100 | 3.7% | 131 | 4.1% | 125 | 4.7% | 0.913 |
| NHPI | 19 | 7.4% | 35 | 12.4% | 34 | 12.0% | 23 | 12.8% | 0.082 |
| AI/AN | **270** | **11.9%** | **368** | **12.9%** | **268** | **14.5%** | **171** | **13.5%** | **0.035** |
| NH white | **1644** | **4.0%** | **1712** | **4.1%** | **1748** | **4.4%** | **1388** | **4.7%** | **<.001** |
| MENA |  |  |  |  |  |  | 18 | 6.4% | **NA** |
| Multiracial | 30 | 13.3% | 34 | 12.3% | 35 | 12.5% | 32 | 10.2% | 0.303 |
| Total | **2687** | **4.9%** | **3034** | **5.1%** | **2947** | **5.2%** | **2416** | **5.6%** | **<.001** |
| ^ Students who checked 3+ racial identities. P values are for linear trends across the 4 surveys  Native Hawaiian/Pacific Islander (NHPI), American Indian/Alaskan Native (AIAN, nHwhite non-Hispanic white (nHwhite), Middle Eastern and North African (MENA)  *Data were collected on MENA for the first time in 2022  ^∆^Data combined across 2 years due to sample size limitations (less than 15 cases per year) | | | | | | | | | |
