# Supplementary material for: Trends and Disparities in Suicidal Thoughts and Behaviors Among an Ethno-Racially Diverse Group of Adolescents: 2013–2022
Source: J Racial Ethn Health Disparities. 2025 May 15;13(3):2452–63. doi: 10.1007/s40615-025-02431-8 (PMC13157415; doi:10.1007/s40615-025-02431-8)
Supplement: Supplementary file 3 — (DOCX 34.6 KB) [file 40615_2025_2431_MOESM3_ESM.docx]

**Supplemental Table 3.** Associations of survey year with past year suicide ideation, by ethno-racial group, odds ratios, unadjusted and adjusted for adverse childhood experiences (ACEs), Minnesota Student Surveys 2013-2022

| **Group / Parameter** | **Unadjusted*** | | **Adjusted**** | |
| --- | --- | --- | --- | --- |
|  | **OR** | **95% CI** | **OR** | **95% CI** |
| **Latine, male** | | | | |
| year 2016 vs 2013 | 1.02 | 0.85-1.22 | 1.05 | 0.87-1.26 |
| year 2019 vs 2013 | **1.38** | **1.16-1.63** | **1.32** | **1.11-1.57** |
| year 2022 vs 2013 | **1.22** | **1.02-1.46** | **1.32** | **1.1-1.59** |
| **Latine, female** | | | | |
| year 2016 vs 2013 | 0.96 | 0.86-1.08 | 0.99 | 0.87-1.12 |
| year 2019 vs 2013 | 1.07 | 0.95-1.2 | 1.01 | 0.9-1.14 |
| year 2022 vs 2013 | **1.21** | **1.07-1.36** | **1.22** | **1.07-1.38** |
| **Black/Somali, male** | | | | |
| year 2016 vs 2013 | 0.88 | 0.52-1.47 | 0.98 | 0.56-1.7 |
| year 2019 vs 2013 | 0.65 | 0.38-1.14 | 0.82 | 0.45-1.49 |
| year 2022 vs 2013 | 0.72 | 0.41-1.26 | 1.12 | 0.61-2.07 |
| **Black/Somali, female** | | | | |
| year 2016 vs 2013 | 0.91 | 0.6-1.37 | 0.94 | 0.6-1.48 |
| year 2019 vs 2013 | 0.67 | 0.44-1.03 | 0.82 | 0.52-1.3 |
| year 2022 vs 2013 | 0.79 | 0.51-1.2 | 1.00 | 0.63-1.58 |
| **Black/Latine, male** | | | | |
| year 2016 vs 2013 | 0.78 | 0.44-1.39 | 0.96 | 0.53-1.75 |
| year 2019 vs 2013 | 0.85 | 0.44-1.64 | 0.91 | 0.46-1.79 |
| year 2022 vs 2013 | 1.06 | 0.56-2.01 | 1.24 | 0.64-2.41 |
| **Black/Latine, female** | | | | |
| year 2016 vs 2013 | 1.11 | 0.75-1.62 | 1.25 | 0.83-1.89 |
| year 2019 vs 2013 | 1.21 | 0.81-1.83 | 1.21 | 0.78-1.87 |
| year 2022 vs 2013 | **1.62** | **1.09-2.41** | **1.82** | **1.18-2.79** |
| **Black/AIAN, male** | | | | |
| year 2016 vs 2013 | 1.08 | 0.71-1.63 | 1.06 | 0.69-1.64 |
| year 2019 vs 2013 | 1.35 | 0.85-2.15 | 1.25 | 0.78-2.03 |
| year 2022 vs 2013 | 1.27 | 0.75-2.15 | 1.51 | 0.87-2.63 |
| **Black/AIAN, female** | | | | |
| year 2016 vs 2013 | 0.86 | 0.67-1.11 | 0.86 | 0.66-1.13 |
| year 2019 vs 2013 | 0.87 | 0.65-1.17 | 0.82 | 0.6-1.11 |
| year 2022 vs 2013 | 1.20 | 0.86-1.69 | 1.03 | 0.72-1.48 |
| **Black, male** | | | | |
| year 2016 vs 2013 | **1.22** | **1.01-1.48** | **1.30** | **1.07-1.58** |
| year 2019 vs 2013 | **1.40** | **1.16-1.69** | **1.44** | **1.19-1.75** |
| year 2022 vs 2013 | **1.42** | **1.17-1.73** | **1.71** | **1.4-2.09** |
| **Black, female** | | | | |
| year 2016 vs 2013 | 0.95 | 0.83-1.08 | 1.00 | 0.88-1.15 |
| year 2019 vs 2013 | 1.08 | 0.95-1.23 | 1.15 | 1-1.31 |
| year 2022 vs 2013 | **1.38** | **1.21-1.56** | **1.52** | **1.33-1.75** |
| **Asian Hmong, male** | | | | |
| year 2016 vs 2013 | 1.09 | 0.82-1.45 | 1.13 | 0.84-1.51 |
| year 2019 vs 2013 | **1.38** | **1.03-1.84** | **1.45** | **1.08-1.95** |
| year 2022 vs 2013 | 1.07 | 0.75-1.53 | 1.21 | 0.84-1.73 |
| **Asian Hmong, female** | | | | |
| year 2016 vs 2013 | **1.66** | **1.34-2.07** | **1.66** | **1.33-2.08** |
| year 2019 vs 2013 | **1.78** | **1.41-2.24** | **1.70** | **1.34-2.16** |
| year 2022 vs 2013 | **2.00** | **1.55-2.57** | **1.98** | **1.53-2.56** |
| **Asian, male** | | | | |
| year 2016 vs 2013 | 1.12 | 0.89-1.4 | 1.16 | 0.92-1.46 |
| year 2019 vs 2013 | **1.44** | **1.16-1.78** | **1.47** | **1.18-1.83** |
| year 2022 vs 2013 | **1.46** | **1.17-1.81** | **1.61** | **1.29-2.02** |
| **Asian, female** | | | | |
| year 2016 vs 2013 | 0.99 | 0.85-1.16 | 1.02 | 0.87-1.2 |
| year 2019 vs 2013 | 0.99 | 0.85-1.15 | 0.99 | 0.84-1.16 |
| year 2022 vs 2013 | **1.30** | **1.12-1.52** | **1.34** | **1.15-1.57** |
| **NHPI, male** | | | | |
| year 2016 vs 2013 | 1.36 | 0.79-2.35 | 1.56 | 0.89-2.73 |
| year 2019 vs 2013 | 1.46 | 0.84-2.54 | 1.49 | 0.84-2.64 |
| year 2022 vs 2013 | **1.77** | **1-3.12** | **1.85** | **1.03-3.33** |
| **NHPI, female** | | | | |
| year 2016 vs 2013 | **1.56** | **1.03-2.35** | 1.45 | 0.94-2.25 |
| year 2019 vs 2013 | 1.42 | 0.93-2.15 | 1.21 | 0.78-1.89 |
| year 2022 vs 2013 | **1.65** | **1.05-2.6** | 1.56 | 0.96-2.52 |
| **AIAN, male** | | | | |
| year 2016 vs 2013 | **1.25** | **1.05-1.49** | **1.30** | **1.08-1.55** |
| year 2019 vs 2013 | **1.53** | **1.26-1.85** | **1.45** | **1.19-1.77** |
| year 2022 vs 2013 | **1.45** | **1.17-1.79** | **1.44** | **1.16-1.78** |
| **AIAN, female** | | | | |
| year 2016 vs 2013 | **1.35** | **1.19-1.53** | **1.38** | **1.21-1.57** |
| year 2019 vs 2013 | **1.50** | **1.3-1.72** | **1.33** | **1.15-1.53** |
| year 2022 vs 2013 | **1.47** | **1.26-1.71** | **1.25** | **1.06-1.47** |
| **NH white, male** | | | | |
| year 2016 vs 2013 | **1.18** | **1.11-1.25** | **1.19** | **1.12-1.26** |
| year 2019 vs 2013 | **1.48** | **1.4-1.56** | **1.44** | **1.36-1.52** |
| year 2022 vs 2013 | **1.41** | **1.33-1.5** | **1.49** | **1.4-1.59** |
| **NH white, female** | | | | |
| year 2016 vs 2013 | **1.21** | **1.16-1.26** | **1.22** | **1.17-1.27** |
| year 2019 vs 2013 | **1.35** | **1.29-1.40** | **1.25** | **1.20-1.30** |
| year 2022 vs 2013 | **1.66** | **1.6-1.73** | **1.55** | **1.49-1.62** |
| **Multiracial 3+, male** | | | | |
| year 2016 vs 2013 | 1.13 | 0.67-1.91 | 1.18 | 0.68-2.06 |
| year 2019 vs 2013 | 1.15 | 0.66-1.99 | 1.34 | 0.75-2.4 |
| year 2022 vs 2013 | 1.07 | 0.62-1.86 | 1.52 | 0.84-2.73 |
| **Multiracial 3+, female** | | | | |
| year 2016 vs 2013 | 1.18 | 0.78-1.77 | 1.23 | 0.79-1.9 |
| year 2019 vs 2013 | 1.20 | 0.8-1.82 | 1.23 | 0.79-1.9 |
| year 2022 vs 2013 | 1.15 | 0.77-1.72 | 1.36 | 0.89-2.1 |

* Unadjusted model included grade as covariate

** Adjusted model included number of ACEs (0, 1-3, 4+, missing)

**Supplemental Table 4.** Associations of survey year with past year suicide attempts, by ethno-racial group, odds ratios, unadjusted and adjusted for adverse childhood experiences (ACEs), Minnesota Student Surveys 2013-2022

| **Group / Parameter** | **Unadjusted** | | **Adjusted** | |
| --- | --- | --- | --- | --- |
|  | **OR** | **95% CI** | **OR** | **95% CI** |
| **Latine, male** | | | | |
| year 2016 vs 2013 | 0.84 | 0.64-1.11 | 0.89 | 0.67-1.18 |
| year 2019 vs 2013 | 1.07 | 0.82-1.39 | 0.98 | 0.75-1.28 |
| year 2022 vs 2013 | 0.95 | 0.72-1.26 | 1.04 | 0.79-1.39 |
| **Latine, female** | | | | |
| year 2016 vs 2013 | 0.90 | 0.77-1.06 | 0.93 | 0.79-1.1 |
| year 2019 vs 2013 | **0.83** | **0.7-0.98** | **0.77** | **0.65-0.91** |
| year 2022 vs 2013 | 0.87 | 0.73-1.03 | 0.85 | 0.71-1.01 |
| **Black Somali, male** | | | | |
| year 2016 vs 2013 | 0.88 | 0.44-1.77 | 1.02 | 0.49-2.11 |
| year 2019 vs 2013 | 0.62 | 0.29-1.33 | 0.82 | 0.37-1.82 |
| year 2022 vs 2013 | 0.80 | 0.38-1.68 | 1.33 | 0.6-2.94 |
| **Black Somali, female** | | | | |
| year 2016 vs 2013 | 0.83 | 0.44-1.54 | 0.87 | 0.45-1.66 |
| year 2019 vs 2013 | 0.79 | 0.43-1.46 | 1.02 | 0.54-1.93 |
| year 2022 vs 2013 | **0.45** | **0.22-0.93** | 0.57 | 0.27-1.2 |
| **Black Latine, male** | | | | |
| year 2016 vs 2013 | 0.65 | 0.3-1.41 | 0.86 | 0.38-1.92 |
| year 2019 vs 2013 | 0.75 | 0.31-1.8 | 0.85 | 0.34-2.11 |
| year 2022 vs 2013 | 0.59 | 0.23-1.52 | 0.73 | 0.27-1.95 |
| **Black Latine, female** | | | | |
| year 2016 vs 2013 | 1.18 | 0.73-1.92 | 1.34 | 0.81-2.24 |
| year 2019 vs 2013 | 1.05 | 0.62-1.79 | 1.00 | 0.57-1.75 |
| year 2022 vs 2013 | 1.30 | 0.78-2.16 | 1.39 | 0.81-2.38 |
| **Black AIAN, male** | | | | |
| year 2016 vs 2013 | 1.20 | 0.59-2.43 | 1.19 | 0.58-2.43 |
| year 2019 vs 2013 | **2.40** | **1.19-4.81** | **2.18** | **1.07-4.43** |
| year 2022 vs 2013 | 2.05 | 0.93-4.53 | **2.49** | **1.1-5.62** |
| **Black AIAN, female** | | | | |
| year 2016 vs 2013 | 0.98 | 0.68-1.4 | 1.00 | 0.69-1.45 |
| year 2019 vs 2013 | 1.12 | 0.75-1.67 | 1.06 | 0.7-1.6 |
| year 2022 vs 2013 | 1.23 | 0.77-1.97 | 1.02 | 0.62-1.66 |
| **Black, male** | | | | |
| year 2016 vs 2013 | 1.22 | 0.91-1.65 | 1.33 | 0.98-1.81 |
| year 2019 vs 2013 | **1.38** | **1.03-1.86** | **1.41** | **1.04-1.9** |
| year 2022 vs 2013 | 1.24 | 0.9-1.7 | **1.50** | **1.09-2.08** |
| **Black, female** | | | | |
| year 2016 vs 2013 | 0.95 | 0.78-1.17 | 1.03 | 0.83-1.27 |
| year 2019 vs 2013 | 1.06 | 0.86-1.29 | 1.11 | 0.9-1.36 |
| year 2022 vs 2013 | **1.37** | **1.12-1.67** | **1.48** | **1.21-1.82** |
| **Asian Hmong, male** | | | | |
| year 2016 vs 2013 | 0.61 | 0.34-1.08 | 0.63 | 0.35-1.12 |
| year 2019 vs 2013 | 1.25 | 0.75-2.09 | 1.29 | 0.77-2.16 |
| year 2022 vs 2013 | 0.62 | 0.3-1.3 | 0.72 | 0.34-1.51 |
| **Asian Hmong, female** | | | | |
| year 2016 vs 2013 | 1.33 | 0.92-1.91 | 1.30 | 0.9-1.88 |
| year 2019 vs 2013 | **1.49** | **1.01-2.18** | 1.36 | 0.93-2.01 |
| year 2022 vs 2013 | 1.41 | 0.91-2.17 | 1.34 | 0.87-2.08 |
| **Asian, male** | | | | |
| year 2016 vs 2013 | 1.09 | 0.72-1.66 | 1.17 | 0.76-1.79 |
| year 2019 vs 2013 | 1.22 | 0.81-1.82 | 1.22 | 0.81-1.84 |
| year 2022 vs 2013 | 0.99 | 0.64-1.52 | 1.12 | 0.72-1.72 |
| **Asian, female** | | | | |
| year 2016 vs 2013 | **0.72** | **0.54-0.94** | **0.73** | **0.55-0.98** |
| year 2019 vs 2013 | 0.81 | 0.63-1.05 | 0.81 | 0.62-1.05 |
| year 2022 vs 2013 | 0.92 | 0.71-1.2 | 0.92 | 0.7-1.21 |
| **NHPI, male** | | | | |
| year 2016 vs 2013 | 1.05 | 0.47-2.37 | 1.38 | 0.59-3.25 |
| year 2019 vs 2013 | 0.74 | 0.29-1.87 | 0.71 | 0.27-1.86 |
| year 2022 vs 2013 | 1.45 | 0.63-3.33 | 1.52 | 0.63-3.66 |
| **NHPI, female** | | | | |
| year 2016 vs 2013 | 1.72 | 0.96-3.08 | 1.58 | 0.86-2.92 |
| year 2019 vs 2013 | 1.65 | 0.92-2.97 | 1.35 | 0.73-2.51 |
| year 2022 vs 2013 | 1.79 | 0.95-3.38 | 1.65 | 0.85-3.22 |
| **AIAN, male** | | | | |
| year 2016 vs 2013 | **1.53** | **1.16-2.01** | **1.63** | **1.23-2.16** |
| year 2019 vs 2013 | **1.67** | **1.23-2.26** | **1.57** | **1.15-2.14** |
| year 2022 vs 2013 | 1.33 | 0.94-1.87 | 1.32 | 0.93-1.88 |
| **AIAN, female** | | | | |
| year 2016 vs 2013 | 1.11 | 0.94-1.31 | 1.11 | 0.93-1.32 |
| year 2019 vs 2013 | **1.28** | **1.07-1.54** | 1.09 | 0.9-1.32 |
| year 2022 vs 2013 | 1.17 | 0.96-1.44 | 0.95 | 0.77-1.18 |
| **NH white, male** | | | | |
| year 2016 vs 2013 | 1.00 | 0.9-1.11 | 1.02 | 0.92-1.14 |
| year 2019 vs 2013 | **1.33** | **1.20-1.47** | **1.25** | **1.13-1.39** |
| year 2022 vs 2013 | **1.12** | **1.00-1.25** | **1.19** | **1.06-1.33** |
| **NH white, female** | | | | |
| year 2016 vs 2013 | 1.03 | 0.96-1.1 | 1.02 | 0.95-1.09 |
| year 2019 vs 2013 | **1.09** | **1.02-1.17** | 0.95 | 0.89-1.02 |
| year 2022 vs 2013 | **1.18** | **1.1-1.27** | 1.01 | 0.94-1.09 |
| **Multiracial, male** | | | | |
| year 2016 vs 2013 | 0.92 | 0.46-1.86 | 0.99 | 0.48-2.04 |
| year 2019 vs 2013 | 0.79 | 0.37-1.7 | 0.89 | 0.41-1.96 |
| year 2022 vs 2013 | 0.47 | 0.2-1.12 | 0.70 | 0.29-1.7 |
| **Multiracial, female** | | | | |
| year 2016 vs 2013 | 0.91 | 0.54-1.54 | 0.94 | 0.54-1.64 |
| year 2019 vs 2013 | 0.96 | 0.57-1.62 | 0.95 | 0.55-1.66 |
| year 2022 vs 2013 | 0.74 | 0.44-1.26 | 0.86 | 0.49-1.5 |

* Unadjusted model included grade as covariate

** Adjusted model included number of ACEs (0, 1-3, 4+, missing)
