# Supplementary material for: Trends and Disparities in Suicidal Thoughts and Behaviors Among an Ethno-Racially Diverse Group of Adolescents: 2013–2022
Source: J Racial Ethn Health Disparities. 2025 May 15;13(3):2452–63. doi: 10.1007/s40615-025-02431-8 (PMC13157415; doi:10.1007/s40615-025-02431-8)
Supplement: Supplementary file 4 — (DOCX 72.9 KB) [file 40615_2025_2431_MOESM4_ESM.docx]

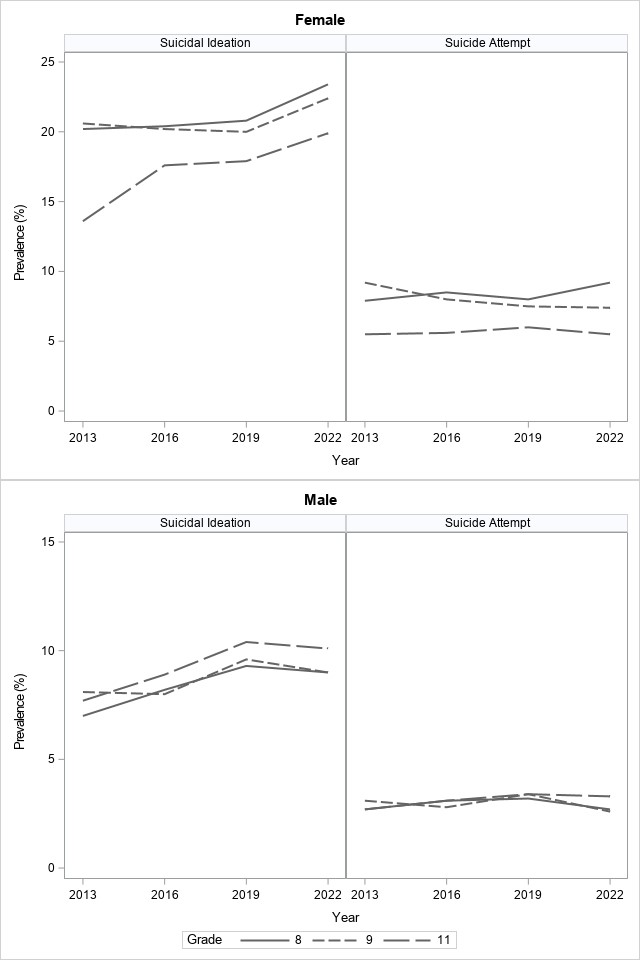


**Supplemental Fig. 1** Prevalence of Suicidal Ideation and Suicide Attempts by Grade and Sex, 2013 -2022 Minnesota Student Surveys (MSS)
